# Guideline “Parkinson’s disease” of the German Society of Neurology (Deutsche Gesellschaft für Neurologie): concepts of care

**DOI:** 10.1007/s00415-024-12546-3

**Published:** 2024-07-06

**Authors:** Lars Tönges, Carsten Buhmann, Carsten Eggers, Stefan Lorenzl, Tobias Warnecke, for the German Parkinson Guideline Group, Lars Tönges, Carsten Buhmann, Carsten Eggers, Stefan Lorenzl, Tobias Warnecke, Mathias Bähr, Jos Becktepe, Daniela Berg, Kathrin Brockmann, Andrés Ceballos-Baumann, Joseph Claßen, Cornelius Deuschl, Günther Deuschl, Richard Dodel, Georg Ebersbach, Thilo Eimeren, Alessandra Fanciulli, Bruno Fimm, Ann-Kristin Folkerts, Madeleine Gausepohl, Alkomiet Hasan, Wiebke Hermann, Rüdiger Hilker-Roggendorf, Günter Höglinger, Matthias Höllerhage, Franziska Hopfner, Wolfgang Jost, Elke Kalbe, Jan Kassubek, Stephan Klebe, Christine Klein, Martin Klietz, Thomas Köglsperger, Andrea Kühn, Paul Krack, Florian Krismer, Gregor Kuhlenbäumer, Johannes Levin, Inga Liepelt-Scarfone, Paul Lingor, Kai Loewenbrück, Matthias Löhle, Sylvia Maaß, Walter Maetzler, Regina Menzel, Philipp T Meyer, Brit Mollenhauer, Manuela Neumann, Per Odin, Tiago Outeiro, Monika Pötter-Nerger, René Reese, Kathrin Reetz, Olaf Rieß, Viktoria Ruf, Anja Schneider, Christoph Schrader, Alfons Schnitzler, Klaus Seppi, Friederike Sixel-Döring, Alexander Storch, Claudia Trenkwalder, Thilo Eimeren, Uwe Walter, Tobias Wächter, Florian Wegner, Christian Winkler, Karsten Witt, Dirk Woitalla, Kirsten Zeuner

**Affiliations:** 1 Department of Neurology, St. Josef-Hospital, Ruhr University Bochum, 44791 Bochum, Germany; 2 Neurodegeneration Research, Protein Research Unit Ruhr (PURE), Ruhr University Bochum, 44801 Bochum, Germany; 3 Department of Neurology, University Hospital Hamburg-Eppendorf, Hamburg, Germany; 4 Department of Neurology, Knappschaftskrankenhaus Bottrop, Bottrop, Germany; 5 Department of Palliative Medicine, University Hospital, Ludwig-Maximilians-University Munich, Munich, Germany; 6 Department of Neurology and Neurorehabilitation, Klinikum Osnabrück-Academic Teaching Hospital of the University of Münster, 49076 Osnabrück, Germany

**Keywords:** Parkinson’s disease, PD day clinic, Multidisciplinary care, PD networks, PD nurse, Palliative care

## Abstract

**Introduction:**

In 2023, the German Society of Neurology published a new guideline on Parkinson’s disease. An important section dealt with PD care concepts, which represent a particularly dynamic field of PD research, including their implementation in clinical practice. Parkinson’s disease is the second most common age-associated neurodegenerative disease. Current estimates of the number of cases in the population describe a significant increase in prevalence in Germany by 2030 with higher proportions in rural areas, which also have a lack of sufficient PD care resources.

**Recommendations:**

In comparison with other international guidelines, which have so far mentioned palliative care and Parkinson’s nurses in particular, the German S2k guideline expands the recommended concepts of PD care to include PD day clinics, inpatient complex treatment, and PD networks.

**Conclusion:**

Concepts of PD care guidelines are necessary because of the complex and rapidly evolving field of PD care provision. If applied appropriately, the potential for optimized care can be exploited and both the patient burden and the economic burden can be reduced. Given that modern care concepts have so far only been applied in a few regions, it is often impossible to generate broad evidence-based data, so that the evaluation of PD care concepts is partly dependent on expert opinion.

## Introduction

This guideline is an abridged and translated short version of the guideline “Parkinson’s disease” of the of the German Society of Neurology (Deutsche Gesellschaft für Neurologie) with its concepts of care sections day-clinic, multidisciplinary care, PD networks, PD nurse, and palliative care. A complete version of this guideline (in German) can be found on the website www.dgn.org/leitlinien and the AWMF (Arbeitsgemeinschaft wissenschaftlicher Medizinischer Gesellschaften) [1]:

AWMF version number: 8.0.

Level of guideline: S2k.

Date of last update: 10/2023.

Valid until: 24.10.2028.

Edited by: Guidelines Committee of the German Neurological Society.

Parkinson’s disease (PD) is the second most common age-associated neurodegenerative disease in Germany. Current estimates of the number of cases in the population of people aged 50 and over amount to 797 prevalent cases per 100,000 and 192 incident cases per 100,000 person-years (validation criteria: multidisciplinary outpatient Parkinson’s diagnosis, inpatient main/secondary diagnosis, and prescribed PD medication) [2]. According to data from the Federal Statistical Office, the number of deaths from primary Parkinson’s syndrome in 2015 was 10,230 [3]. Due to demographic change and the associated aging of society, age-related diseases such as PD will continue to increase, with an estimated 36% increase in the prevalence rate in Germany between 2005 and 2030 [4]. This trend will become a challenge for rural areas in particular, which are characterized by a higher proportion of older people, in terms of nationwide services and structures for high-quality long-term care for PD patients. Treatment in Germany has already required more and more resources in recent years and requires close coordination between outpatient and inpatient treatment sectors [5, 6].

In the outpatient sector, the number of doctor contacts per PD patient (Ø 15.2 contacts per year) is already significantly higher compared to patients without PD (Ø 12.2 contacts per year) and this results in more drug prescriptions (37.7 compared to 21.7 prescriptions per year). On average, PD patients are hospitalized twice as often (once per year) as non-PD patients (0.5 times per year) and the length of stay is twice as long (9.8 days compared to 4.8 days) [7]. From 2010 to 2015, the number of hospitalized patients in Germany with a main diagnosis of PD disease increased significantly by 27% from 33,760 cases to 42,980 cases per year. More than one PD patient per 1000 inhabitants had to be hospitalized at least once a year, which placed a heavy burden on local care structures [6]. The guideline “Parkinson’s disease” of the German Society of Neurology [1] contains a concepts of care section on these issues, which is presented here.

## What is new?

Novel strategies and evaluations of care concepts include PD day clinic (PDC), inpatient PD multimodal complex treatment (PD-MCT), integrated PD care networks, and palliative care.

## The most important recommendations

- Patients with insufficiently diagnosed Parkinsonian syndromes can be admitted to a PD day clinic for differential diagnostics.
- PD patients can be admitted to a PD day clinic for complex drug therapy adjustments, pre-examinations, initiation, and follow-up examinations of invasive procedures such as pen or pump therapies or deep brain stimulation, which cannot be implemented on an outpatient basis.
- Inpatient multidisciplinary therapy (e.g., multimodal complex treatment, PD-MCT) should be used in preference to standard inpatient therapy for PD.
- It can be recommended that patients are treated in PD networks as part of an integrated care approach.
- It can be recommended that people with PD have regular access to care from a specialist PD nurse.
- Principles for palliative care (both outpatient and inpatient) comprise access to a multidisciplinary team including a physiotherapist, occupational therapist, speech therapist, psychologist, social worker, nurse, and doctor. The utilization of a PD multidisciplinary team can lead to an improvement in quality of life.

## Guidelines in detail

### PD day clinic

#### Patient selection

The concept of day clinic care for PD patients is still relatively new and had been applied only in more general terms in German clinics before. There is no standardized definition for which patients should be treated in a PD day clinic (PDC). A multidisciplinary therapeutic approach is generally recommended for PD patients [8, 9]. PD patients should be treated in a PDC if their clinical care in the outpatient setting is too complex or too costly in terms of personnel, time, or expertise, but for various reasons inpatient treatment is not appropriate, possible, or desired [5, 10, 11]. These are patients who are on the borderline between inpatient and outpatient care due to an increased need for differentiated and/or comprehensive treatment strategies.

Criteria which, when present alone or in combination, make treatment in a PDC appear recommendable are [5, 10, 11]:

- Patients with relevant motor and/or non-motor symptoms.
- Patients with complex medication regimens and/or extensive therapy regimens.
- Patients with invasive therapy measures such as medication pumps (apomorphine, Duodopa) or deep brain stimulation (DBS).
- Patients for whom the effects of treatment adjustment should be evaluated over a longer period of time at longer intervals than is possible on an inpatient basis, but more closely than on an outpatient basis.
- Differentiation of the indication for invasive therapy (apomorphine or levodopa pump, THS).
- Interval checks after DBS implantation.
- Patients with cognitive deficits who have shown psychotic or delirious symptoms during previous inpatient stays or overnight stays away from home in everyday life.
- Patients who are unable to stay in hospital for private reasons (work, caring for relatives, looking after animals, etc.).
- Patients with an etiologically unclear PD diagnosis.

Therapeutic measures that make treatment in the PDC recommendable:

- Relevant changes or adjustments to complex medication regimens.
- Dose adjustments for pump therapies.
- New installation of an apomorphine pump.
- Adjustment to an apomorphine pen.
- Contact testing, reprogramming, or reprogramming for deep brain stimulation (DBS).
- Symptom-oriented activating therapies (physiotherapy, occupational therapy, speech therapy).
- Creation of customized oral and written therapy concepts for outpatient activating therapies.

Diagnostic measures that make treatment in the PDC recommendable:

- Standardized levodopa test.
- Apomorphine test.
- Comprehensive neuropsychological testing.
- Swallowing diagnostics (FEES).
- Differentiation of the indication for activating therapies (physiotherapy, occupational therapy, speech therapy) and/or review of the objectives of previous outpatient therapy procedures.

Patients who are not considered suitable for treatment in the PDC:

- Mildly affected PD patients who can be adequately treated on an outpatient basis.
- PD patients who require inpatient treatment.
- Severely affected and/or multimorbid patients.
- Immobile patients.
- Patients with severe dementia.
- Patients with unreasonably long travel distances.
- Patients with pronounced psychotic symptoms.

**Table:** 

Recommendation-specific supportive text	
The following patient groups can be treated in the PDC Patients with unclear parkinsonian syndromes can be admitted to a PDC for differential diagnostics and PD patients for complicated medication therapy adjustments, pre-examinations, initiation, and follow-up examinations of invasive procedures such as pen or pump therapies or deep brain stimulation, which cannot be carried out on an outpatient basis In addition, treatment in a PDC can be offered if The effects of treatment adaptation should be evaluated over a longer period of time at greater intervals than inpatient treatment, but more closely than is possible in an outpatient setting Inpatient treatment appears useful in principle, but this is not suggested (e.g., risk of nocturnal delirium in cognitively impaired patients) or possible (e.g., occupational, caring for relatives) for individual reasons	

#### What are the differences between treatment in a day clinic from inpatient care treatment?

In general, the treatment of PD patients in outpatient, day clinic, and inpatient settings is not standardized and the services as well as competencies of the providers are not precisely defined. Accordingly, there are considerable geographical differences in the care structure. PD patients receive outpatient care at university hospitals, sometimes in specialized outpatient clinics or medical care centers, while in certain regions there are specialist PD practices in private practice. While remuneration in the university polyclinics is regulated by a university rate, remuneration for medical services in the private practice sector is based on German EBM or GOÄ charges.

A large proportion of patients are treated by registered neurologists or neurologists and general practitioners. The exact figures on the share of individual providers in the care of patients are not available. Day clinic structures are becoming increasingly important and are currently mainly found at university clinics, but also at clinics with special expertise in PD. Billing is based on a special daily rate, which must be negotiated with the health insurance companies. PD patients are usually treated as inpatients in the hospital responsible for acute medical care and paid according to the German DRG system. In the case of special diagnostic or therapeutic issues, treatment usually takes place in an inpatient facility with special PD expertise.

In a review [8], it is discussed that multidisciplinary care is essential for the treatment of PD, but that broad implementation is limited for a number of reasons. Expert opinion suggests team-based care that is decentralized and integrated vertically and horizontally across health systems. Treatment in PDC should combine the treatment benefits of inpatient and outpatient settings [6]. In the PDC, treatment takes place in a multi-professional team in which the PD nurse plays a key role [5].

Differences between day clinic and standard outpatient treatment are:

- Better option of carrying out patient-centered therapy by considering the individual motor and non-motor subtype of PD [12].
- More time, personnel, and equipment capacities.
- Specialist expertise compared to non-PD specialty practices.
- Multi-professional team on site.
- Possibility to observe the patient for several hours a day and thus directly assess therapeutic changes, recognize motor and non-motor fluctuations and dyskinesias during the course of the day, and objectify the often uncertain anamnestic information and perceptions [13].
- Possibility of presenting the patient with various therapeutic options that have been demonstrated to improve motor and non-motor functions [14] and motivating them to use these in the outpatient setting (e.g., stress management, autogenic training, yoga, tai chi, LSVT® BIG, music therapy).
- Educational measures for patients and relatives can be provided several times, which is often necessary to achieve and maintain successful treatment.
- Addressing the frequently existing psychosocial and/or sociomedical problems [15], which often cannot be adequately discussed and clarified in the outpatient setting, primarily due to time constraints.
- Reimbursement via a daily rate to be negotiated with the health insurance funds in the PDC instead of via self-payers/insured/insured or university outpatient rates.

Differences between day clinic and standard inpatient treatment are (Table 1):

- Structural characteristics are defined for the PD-MCT [https://www.dimdi.de/static/de/klassifikationen/ops/kode-suche/opshtml2019/block-8-97...8-98.htm]. Criteria are primarily focused on quantitative aspects (therapy frequency and duration) and less on qualitative aspects (general neurological and non-Parkinson’s-specific expertise of the service providers, equipment).
- Therapy adaptation under everyday conditions instead of in an inpatient environment with different challenges and daily routines that are not comparable to everyday life.
- Individually (patient- and/or facility-related) selected intervals between treatment days.
- Overall, prospectively longer observation period of the patient (possibly over weeks).
- Therapy setting under everyday conditions, as the patients are exposed to their usual daily stress factors (job, social life, usual day/night rhythm, as well as times for meals and taking medication) in the days between the stays in the PDC at home.
- No limitation of treatment to an average length of stay of the PD diagnosis-related case grouping (DRG).
- No nocturnally altered environment, which can lead to delirious symptoms with the risk of increased inpatient mortality during inpatient stays, particularly in the case of dementia, older patients, and patients with more severe disease.
- Remuneration via a daily rate to be negotiated with the health insurance funds in the PDC instead of via diagnosis-related groups (DRG).

**Table 1:** Differences between various PD care concepts

	PD day clinic (PDC)	PD complex treatment (PD-MCT)	PD specialist practice
Indication	PD and atypical parkinsonian syndromes		
Setting	Day clinic, semi-inpatient without overnight stay	Full inpatient with overnight stay	Outpatient
Target group	Patients with the need for intensified therapy adaptation under everyday stress who fall into a care gap between outpatient and inpatient treatment	Patients with subacute, crisis-like conditions or progressive clinical deterioration under outpatient conditions	Patients in the early and later stages of the disease who should or must be continuously cared for with special expertise during the course of the disease
Arrival	Independently or with help Rather close to home	Independently or with help Rather independent of place of residence	Independently or with help Rather close to home
Stay	Intermittent, e.g., on 5 days within 3 weeks, additional days if necessary	14–21 days depending on the clinic, possibly extended by 1 week	At regular intervals, often quarterly
Remuneration	Daily rate individually negotiated with health insurance companies	DRG or OPS 8-97d for PKB after successful structural review by medical service	EBM or GOÄ catalog
Concept	Multimodal, interdisciplinary	Multimodal, interdisciplinary	Cooperative and networked
Alignment	Therapy optimization with a focus on adapting complex drug therapy regimens and/or settings of drug pumps or deep brain stimulation	Therapy optimization with a focus on training	Therapy optimization with a focus on continuous care, including patients with invasive therapies (THS, pumps)
Involvement of non-medical staff	Yes (several areas from speech therapy, occupational therapy, physiotherapy, social services, nutritional counseling, music therapy)	Yes (at least 3 areas from sports therapy, speech therapy, psychotherapy, and art/music therapy)	Provision of a PD assistant (PASS) mandatory
Therapy time per week	Individual timetable, at least 5 h/day, including at least 1 medical contact in an individual setting (30 min)	At least 7½ h, of which at least 5 h in individual therapy	Not specified
Team meeting	Daily between doctor and PD nurse, weekly in the team	Weekly	Not specified
Documentation	Daily (treatment goals and results)	Weekly (treatment goals and results)	Not specified
PD nurse	Mandatory	Desirable	Not specified
Certification criteria	Primarily qualitative (https://www.parkinson-vereinigung.de/files/Kliniken%20Parkinson/Checkliste%20Parkinson%20TK_Audithilfe_cb_cf.pdf)	Primarily quantitative (https://www.parkinson-vereinigung.de/diverse-inhalte/fachkliniken/zertifizieungskriterien.html)	Quantitative and qualitative (https://www.parkinson-vereinigung.de/files/Kliniken%20Parkinson/Checkliste%20Parkinson%20SP_Audithilfe.pdf)
Evidence of effectiveness	Evidence level 2 16-month outcome [4] 32-month outcome [5]	Not available	Not available

In contrast to inpatient treatment, treatment in a day clinic is limited to patients who live within a reasonable distance or time from the day clinic. This considerably limits the number of potentially suitable patients.

**Table:** 

Recommendation-specific supportive text	
Partial inpatient treatment, intermittently frequent, multidisciplinary treatment that can be flexibly determined in terms of time interval and frequency Availability of clinical short-term outcome data In a PDC, health insurance companies can be billed as a daily rate according to an individual treatment concept defined by the treatment center Therapeutically complex, still sufficiently mobile patients with the need for intensified and controlled therapy adaptation under everyday stress Therapeutic focus on adaptation of complex drug therapy regimes and/or settings of drug pumps or deep brain stimulation In contrast to inpatient treatment, intensified therapy adaptation under everyday stress should be carried out in PDC; in contrast to outpatient treatment, monitoring of complex oral therapy regimens, pump therapy, or after DBS should be carried out	

#### What is the optimal treatment period and the optimal treatment intensity in a PDC?

Treatment concepts in the still few PDC sin Germany vary from daily treatment over a short period of time to treatment days at longer intervals over longer periods of time, both between the individual facilities and in relation to patients within the facilities. In general, the concept of a PDC with fixed daily rates offers the possibility of customizing treatment periods and treatment intensities to the individual patient. To date, there are no comparative studies on different treatment periods and treatment intensities in a PDC. The authors suggest the Hamburg model as a recommendable concept with a treatment period of 3 weeks and a treatment intensity with therapy on 5 days (1st week: Monday and Thursday, 2nd week: Wednesday, 3rd week: Tuesday and Friday), as objective and subjective therapy successes have been evaluated for treatment according to this concept [10, 11]. In certain cases, a patient-specific deviation from this concept in terms of treatment duration and intensity also appears to make sense in terms of both the institution and the patient.

**Table:** 

Recommendation-specific supportive text	
The treatment period and treatment intensity in a PDC should run for at least 3 weeks, with a total treatment intensity of 5 days and a respective interval of 3–6 days Treatment duration and intensity can be modified individually if necessary	

#### How effective is therapy in a day clinic compared to standard outpatient treatment in the treatment of PD?

To date, there are no published comparative studies on the effectiveness of a defined day hospital setting for PD patients compared to a defined standard outpatient treatment.

#### How effective is therapy in a day clinic compared to standard inpatient treatment in the treatment of PD?

For both PD complex treatment [16, 17] and the therapeutic concept of the day clinic according to the Hamburg model [10, 11], it has been shown that motor and non-motor symptoms could be improved at the end of therapy compared to the beginning. However, no data are yet available comparing the effectiveness of treatment in a day clinic setting with that in an inpatient setting, including specialized inpatient PD complex treatment.

### Inpatient multidisciplinary therapy

#### How effective is inpatient multidisciplinary therapy (e.g., multimodal complex treatment) compared to standard inpatient therapy in the treatment of PD?

Inpatient multidisciplinary therapy for PD is carried out in Germany as PD multimodal complex treatment (PD-MCT) or PD complex therapy (PCT) in more than 200 hospitals [18]. Here, pharmacological therapy is used together with non-pharmacological therapies. These include physiotherapy and occupational therapy as well as speech therapy, sports therapy, or artistic therapies that are also referred to as activating therapies [19]. In Germany, PD-MCT is used for at least 7, but usually 14–20 days [18]. From a medical perspective, the aim of the therapy is to optimize functional ability, reduce disability, and thereby improve quality of life. Internationally, inpatient multidisciplinary therapies with different designs have been able to improve the motor symptoms, everyday activities, and quality of life of patients with PD in randomized controlled trials. Some of the effects lasted up to 1 year after the end of the intervention [20, 21]. There are no direct comparative studies on the effectiveness of inpatient multidisciplinary therapy compared to standard inpatient therapy in the treatment of PD. It should be noted that there are no defined criteria for "standard inpatient therapy", so that a comparison would be methodologically difficult. However, it can be assumed that inpatient multidisciplinary therapy is clearly superior to the usual inpatient care due to the defined minimum scope of non-pharmacological activating therapies.

**Table:** 

Recommendation-specific supportive text	
Inpatient multidisciplinary therapy (e.g., multimodal complex treatment) should be prioritized over standard inpatient treatment of PD	

### PD care networks

#### How effective is integrated care in a care network compared to standard outpatient therapy in the treatment of PD?

PD networks are regional care approaches for improving the care of PD patients through intensified cooperation between professional groups, more intensive exchange, improved communication, knowledge transfer, and further training [3]. International data on integrated care in the sense of a coordinated care process show that such care coordination can achieve an improvement in Parkinson’s-related quality of life [22]. In Germany, there is no nationwide distribution of PD networks, so that so far it has always been a matter of regional and supra-regional initiatives with different focus settings. Current data on PD care networks demonstrate overall that an integrated treatment model involving a care coordinator over a period of 6 months has led to a significant improvement in health-related quality of life. Here, studies with a treatment period of at least 6 months and in the outpatient sector were significantly superior to studies with a shorter treatment period and/or in the inpatient sector. It is also evident that network structures can improve communication and cooperation between professional healthcare providers in Germany. Based on a social network analysis from the PD Network Münsterland, it became clear that regular exchange in plenary meetings, structured communication paths, and further training lead to an increased transfer of knowledge as well as increased professional communication and specialization [23].

**Table:** 

Recommendation-specific supportive text	
It can be recommended that patients are treated in PD networks as part of an integrated care approach	

### PD nursing care

#### How effective is care by a specialized PD nurse (PN) compared to standard medical therapy in the treatment of PD?

Specialized nurses for patients with PD, so-called PD nurses (PN), exist for more than 40 years, including in England, the USA and the Scandinavian countries. However, no uniform international training standard for PNs has yet been developed. The working conditions in the national and regional healthcare systems are very different. In England, PNs sometimes work together with neurologists or geriatricians, but often also with GPs or independently, sometimes in hospitals, but often in private practices. In Germany, most PNs work in hospitals and under the supervision of a neurologist. The work tasks of PNs vary between the different workplaces and can include, for example, case management tasks, care of patients with complex forms of therapy such as pen/pump therapies and deep brain stimulation, or clinical research tasks. In Scandinavia and the Netherlands, a PN is often part of a multi-professional "PD team", which also includes physicians specializing in Parkinson’s, physiotherapists, occupational therapists, and speech therapists. In Germany, this type of treatment team is still rare in the outpatient setting, but is increasingly being offered in PD networks. Overall, there is still relatively little evidence on the effect of PN intervention in patients with PD. The results to date reflect only limited benefits in terms of clinical symptomatology when PN care is offered in addition to the treating GP or neurologist. The results show that sufficient expertise and capacity for monitoring, medication adjustment, information, and support are essential and can be provided by different professional groups.

**Table:** 

Recommendation-specific supportive text	
It may be recommended that people with PD have regular access to care from a PN The PN may be involved in the clinical monitoring and adjustment of medication and the monitoring of deep brain stimulation in consultation with the treating physicians The PN may be in regular contact with carers, including home visits where appropriate The PN may be available as a reliable source of information on clinical and social issues affecting PD patients and their carers/families	

### Palliative care

#### What specific symptoms occur in the final stage of the disease compared to the previous course of the disease?

To date, there is no standardized definition of the palliative phase of PD. In a current definition, a Hoehn & Yahr stage of III or higher together with a loss of autonomy of the patient is considered a palliative intervention stage [24, 25]. On the other hand, the late stage of PD disease is defined with a disease duration of at least 7 years and a Hoehn and Yahr stage of at least IV or profound limitations in the activities of daily living [26]. The final stage of PD is defined by advanced motor PD with significant limitations in autonomy or independence in activities of daily living. Patients show complex motor fluctuations and suffer from severe neuropsychiatric symptoms in addition to other non-motor symptoms.

**Table:** 

Recommendation-specific supportive text	
It can be extrapolated from the available data that the palliative phase of PD is characterized by loss of autonomy and a very high symptom burden that neuropsychiatric symptoms in particular play an important role in this phase of the disease and contribute to poor quality of life that swallowing disorders, delirium, and aspects associated with polypharmacy play an important role in the management of patients	

#### What specific drug and non-drug symptom control is possible in the final stage of the disease compared to the standard treatment of PD?

PD patients in the late stages of the disease are highly dependent on activities of daily living and have considerable medical needs. There is a considerable burden on family caregivers and professional caregivers as well as a greater economic burden on families and the healthcare system. In the late stages, the clinical picture is increasingly characterized by non-motor symptoms. In this phase of PD, oral intake of medication is often difficult or delayed. This restricts the administration of medication for symptom control. On the other hand, the cognitive changes mean that non-drug therapy methods are only possible to a limited extent. The increasing restriction of mobility often leads to secondary complications such as fractures or pneumonia.

**Table:** 

Recommendation-specific supportive text	
The following therapeutic options can be offered: Application of levodopa with preserved swallowing ability or via a PEG tube; an alternative, particularly in the case of dysphagia or ileus symptoms, is the administration of rectally administered, rapidly soluble levodopa Use of the rotigotine patch in low doses, especially for swallowing disorders Application of apomorphine s.c. (application with initially low-dose pump therapy, no single injections, as palliative care should involve as little manipulation of the patient as possible) Continuation of stimulating physiotherapeutic and neuropsychological therapy measures	

#### What palliative outpatient or inpatient care do patients and relatives with PD need in the final stage of the disease?

The palliative phase of PD has been little studied to date. In recent years, however, there has been a paradigm shift in the definition of the area of application. Palliative care is not only aimed at alleviating suffering in the final stage of the disease, but also considers earlier phases of the disease. The palliative care approach is not limited to pain relief, but also addresses all physical, psychological, and spiritual problems that worsen the quality of life during the course of the illness. The term "early integration" coined in this context encompasses the early inclusion of palliative structures and can be helpful in coping with the illness and maintaining quality of life. The exact point at which palliative care is initiated is unclear. The physical and emotional burden on patients and their families increases significantly during this phase, as do the direct and indirect costs of illness. Most relatives see it as their duty to care for the seriously ill patient at home, often unaware of the social support options available.

The following principles can be applied to palliative care (both outpatient and inpatient):

- Patients should have access to a multidisciplinary team consisting of physiotherapy, occupational therapy, speech therapy, psychologists, social workers, nurses, and doctors. The use of a multidisciplinary team can lead to an improvement in quality of life.
- Communication with patients and relatives should be open and structured and include specific treatment goals and options.
- It is highly recommended to consider advanced care planning early in the disease process, especially before the development of cognitive deficits.
- Lasting powers of attorney can be helpful in care planning, but are often too unspecific with regard to Parkinson’s-specific care content. Structured advice and information about options and content should be provided by the attending physician.

A multi-professional team can provide relief for the patient, but above all for the family carers. Care in an inpatient care facility can also provide relief. It is very important that a detailed symptom analysis is carried out in the late phase of PD. A multi-professional team should bring together the needs of the patient and relatives and accompany the natural dying process by providing the best possible symptom control. A detailed power of attorney tailored to the patient’s condition helps to specify the patient’s individual wishes for the last phase of life. A pacemaker unit for deep brain stimulation can also be replaced in the late phase of the disease.

**Table:** 

Recommendation-specific supportive text	
It may be considered: That a power of attorney with specific Parkinson’s-related content is drawn up An inpatient nursing home admission can serve to relieve the patient and relatives A deep brain stimulation unit can also be replaced in the late phase of the disease, particularly in the presence of deep brain stimulation withdrawal syndrome A multi-professional team should work together to meet the individual needs of the patient	

## Conclusion

In comparison with other international guidelines on the same topics, this German S2k guideline significantly expands the recommended concepts of care to include day clinics, inpatient complex treatment, and PD networks in addition to palliative care and PD nurses. The efficacy of all interventions depend on many variables such as patient disease burden and caregiver burden, but also on geographical availability and the solution of logistical challenges. The decision on the application of these interventions, their development, and billing by insurance companies will also be influenced by socio-economic policy decisions in the mid- and long erm. However, in this guideline manuscript, the focus was on a primarily medical/neurological perspective, which should be integrated into broader discussions including socioeconomic issues. Also from a broader perspective, this manuscript emphasizes the increasing importance of interdisciplinary care concepts for the management of PD, with the goal of enabling wider implementation and easier access for those affected.
